# A Comparative Analysis of *Weizmannia coagulans* Genomes Unravels the Genetic Potential for Biotechnological Applications

**DOI:** 10.3390/ijms23063135

**Published:** 2022-03-15

**Authors:** Martina Aulitto, Laura Martinez-Alvarez, Gabriella Fiorentino, Danila Limauro, Xu Peng, Patrizia Contursi

**Affiliations:** 1 Dipartimento di Biologia, University of Naples Federico II, 80126 Naples, Italy; martina.aulitto@unina.it (M.A.); fiogabri@unina.it (G.F.); limauro@unina.it (D.L.); 2 Biological Systems and Engineering Division, Lawrence Berkeley National Laboratory, Berkeley, CA 94720, USA; 3 Archaea Centre, Department of Biology, University of Copenhagen, DK-1165 Copenhagen, Denmark; laura.martinez@bio.ku.dk (L.M.-A.); peng@bio.ku.dk (X.P.); 4 BAT Center—Interuniversity Center for Studies on Bioinspired Agro-Environmental Technology, University of Naples Federico II, 80055 Portici, Italy; 5 Task Force on Microbiome Studies, University of Naples Federico II, 80126 Naples, Italy

**Keywords:** *B. coagulans*, CAZymes, bacteriocins, CRISPR-Cas systems

## Abstract

The production of biochemicals requires the use of microbial strains with efficient substrate conversion and excellent environmental robustness, such as *Weizmannia coagulans* species. So far, the genomes of 47 strains have been sequenced. Herein, we report a comparative genomic analysis of nine strains on the full repertoire of Carbohydrate-Active enZymes (CAZymes), secretion systems, and resistance mechanisms to environmental challenges. Moreover, Clustered Regularly Interspaced Short Palindromic Repeats (CRISPR) immune system along with CRISPR-associated (Cas) genes, was also analyzed. Overall, this study expands our understanding of the strain’s genomic diversity of *W. coagulans* to fully exploit its potential in biotechnological applications.

## 1. Introduction

Wide substrate efficiency and excellent environmental robustness are indispensable features for the microbial-based production of biochemicals [1,2]. For instance, bacterial fermentation is currently used as an eco-sustainable alternative to obtain lactic acid (LA) from raw materials [3]. So far, several lactic-acid producer strains of *W. coagulans* have been used for the production of LA from lignocellulosic biomasses [4,5,6]. Interestingly, *W. coagulans* grows at temperatures ranging from 50 to 55 °C and pH 5–6 that are chemico-physical conditions comparable to those used for the biomass saccharification [7,8,9]. Therefore, *W. coagulans* represents an attractive biocatalyst for LA production because of its thermophilic nature that not only reduces the risk of microbial contamination but also makes it possible to develop efficient fermentation processes, i.e., simultaneous saccharification and fermentation (SSF) [8,10]. Recently, a novel *W. coagulans* strain, designed as MA-13 and isolated from canned beans manufacturing, has been tested in an SSF configuration for efficient production of LA from wheat straw [7]. Moreover, it turned out to be exceptionally resistant to extreme conditions, such as the presence of toxic compounds derived from the thermo-acidic treatment of lignocellulose pointing to this microbe as a suitable biological tool for the eco-friendly production of LA [8].

*W. coagulans* is a homofermentative microorganism, which produces LA from glucose via the Embden–Meyerhof–Parnas (EMP) pathway and from xylose via the pentose phosphate pathway (PPP) or the phosphoketolase pathway (PKP). Specifically, the yield of LA varies from 60 to 98% of the total fermentation products depending on whether xylose is metabolized through one or the other pathway [4]. Interestingly, genomic comparison among different *W. coagulans* strains have revealed that some of them lack at least some of the genetic determinants of the pentose phosphate pathway, hinting at a significant metabolic and genomic diversity among the isolates [4,9,11]. On the other hand, knowledge of the genomic features of the different *W. coagulans* strains is essential to fully exploit their potential in the white biotechnology applications [12]. Indeed, *W. coagulans* is also emerging as a promising GRAS (Generally Regarded As Safe) probiotic candidate, by sharing characteristics with both *Bacillaceae* and *Lactobacillaceae* [13]. Currently, *W. coagulans* strains are widely used as probiotics in marketed worldwide formulations namely, Sustenex, Lactospore, Neolactoflorene, etc. [14]. In a recent study, it has been demonstrated that MA-13 over-produces under standard growth conditions α- and β-galactosidases that are key enzymes for improving the nutritional value of lactose-containing food as well as for the production of galactooligosaccharides (GOS) that improve the digestibility and nutritional value of food. Moreover, the identification and comparative analysis of the full repertoire of *W. coagulans* CAZymes might help to shed light on the probiotic and prebiotic properties of the available strains [15]. Furthermore, *W. coagulans* has been also exploited in other diverse biotechnological applications such as in medicine, food, and chemical industry including the production of thermostable enzymes and antimicrobial peptides, such as coagulin [16]. Likewise microorganisms isolated from food fermentation or spoilage, Firmicutes display a significant variation within a given species, especially for gene clusters involved in the fitness or adaptation to specific conditions [17]. So far, many *W. coagulans* strains have been isolated and the genome sequences of 47 and 24 are available on National Center for Biotechnology Information (NCBI) and Integrated microbial Genomes (IMG), respectively (1 February 2022) [9,18,19,20]. To fully explore the biotechnological potential of MA-13, a comparative analysis among the 9 selected strains has been carried out. Genome decoding is functional to unravel molecular mechanisms driving diversification, genetic variability, and host–pathogen interactions [21]. Previously, a comparative genomic analyses on a few *W. coagulans* strains with a special focus on carbohydrates catabolism and genetic determinants responsible for general defense systems from foreign DNA has been reported [4]. In our analysis, we picked up the closest evolutionary related strains to MA-13 and pinpointed the core, the dispensable genes that are present in some but not all the strains studied (non-core) as well as the strain-specific genes (singletons) related to CAZymes, protein secretion systems, antimicrobial peptides/proteins, secondary metabolites, and the defense systems protecting cells from foreign DNA. Moreover, we show that the genomic content varied among close individual strains, and such variations are traced back to the gain or loss of singleton, genomic islands, selfish DNA (plasmids, bacteriophages, integrative conjugative elements), and/or widespread horizontal gene transfer (HGT) [22].

## 2. Results and Discussion

### 2.1. Genomic Features of W. coagulans Strains

A draft of the MA-13 genome was recently published [23] supplying a general overview of its genetic content. In the current work, we provide a comparative analysis among phylogenetically related strains, with the purpose to highlight the genomic features that indicate potential in different biotechnological fields. To this aim, the genome sequence of MA-13 was re-assembled using different tools (data not shown) and according to the quality check (QUAST), the best result was obtained using MEGAHIT. The updated genome assembly was deposited in NCBI under the same accession number (WGS: SMSP02000001-SMSP02000116, ACCESSION SMSP00000000) and consists of 116 contigs, with a total length of 2,942,169 bp and an average GC% content of 47.2%. Compared to the previous work [23], the number of contigs was drastically reduced (from 1653 to 116 contigs) and the total length adjusted from 3,237,270 to 2,942,169 bp. To date, 47 *W. coagulans* assemblies have been deposited in the NCBI database and their genomic features are summarized in Table S1. Only 13 assemblies were reported to be sequenced as complete genome, while the others are deposited as draft. In general, *W. coagulans* genome size varies from 2.86 to 3.69 Mbp with an average size set around 3.33 Mbp as described for MA-13 as well as for H1, XZL4, AF24-21, AF24-19, MGYG-HGUT-00191, B4098, DSM1 strains (Table S1). The number of coding sequences ranges from 2555 to 3237 and the GC% content is around 46–47% (Table S1).

### 2.2. Phylogenetic and Comparative Genomic Analysis

In microbial genomics, phylogenetic analysis is crucial not only to establish the genetic novelty and the genotype–phenotype relationships of the isolates but also to identify the closest relatives within assembled genomes. With this aim, a species tree was generated using the genomes of nine *W. coagulans* strains available in the KBase system.

**Figure 1:**
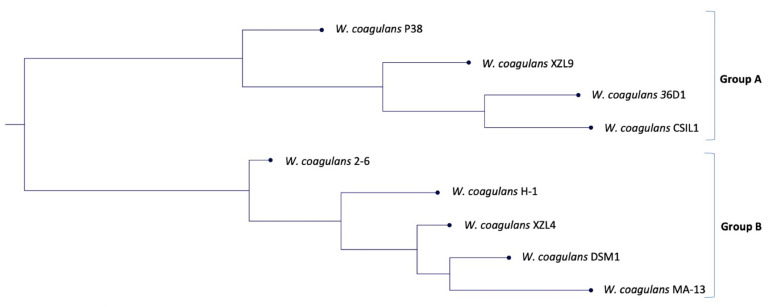
Phylogenetic tree based on the genome comparison of the nine strains of *W. coagulans*.

In our previous work, a phylogenetic tree was built based on 16S rRNA genes and two main groups were identified [7]. In this study, the comparative whole-genome sequence analysis highlighted the presence of two clades (named Group A and B) that underline the coexistence of two distinct evolutionary lineages. This hypothesis is supported by the similar genome size within each clade (~2.9 Mbp for Group A and ~3.4 Mbp for Group B, respectively) (Figure 1, Table S1). Moreover, the closest strain to MA-13 is DSM1 (accession number NZ_CP009709) which was also isolated from agri-food wastes [20]. Then, we resolved to calculate the average nucleotide identity (ANI) value of orthologous genes shared by the two genomes. The FastANI analysis of the reciprocal mappings shows the close evolutionary relationship between the two strains (MA-13 and DSM1) with an ANI value of 98.43% (Table 1).

Moreover, as further evidence of the evolutionary connection, we have calculated the digital DNA–DNA hybridization (dDDH) value using the recommended Formula 2 of the Genome-To-Genome Distance Calculator (GGDC) [24]. The estimated DDH yield was 87.70%, with a probability that these strains belong to the same species and subspecies of 94.96% and 60.49% (via logistic regression), respectively.

**Figure 2:**
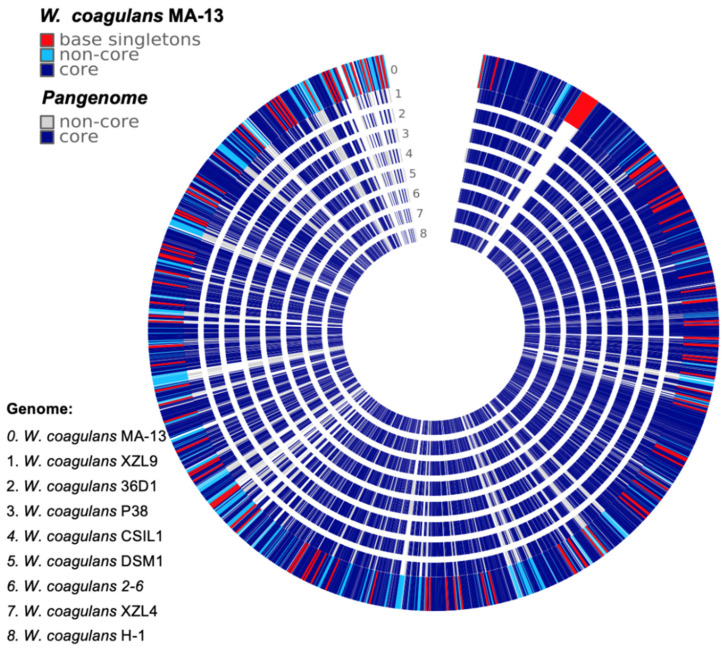
Comparative genomic analysis of *W. coagulans* MA-13 and the closest eight relatives.

To get a deeper insight into the genomic variation among the nine strains a comparison of all the genes grouped by sequence homology was carried out [25]. This analysis was performed using the phylogenetic tree (Figure 1) as input to build up a framework for exploring the genomic diversity and the common features through the analysis of non-core and core genes, and singletons (Figure 2, Table S2). The non-core set is represented by all genes not universally conserved within a species, whilst the core is constituted by genes conserved across all the genomes [26].

**Figure 3:**
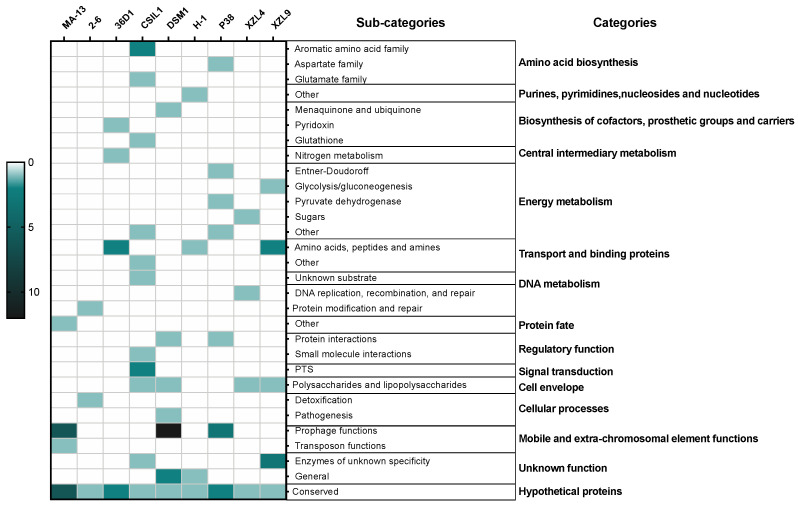
Heat-map of singletons. The depth of color corresponds to the number of proteins. Dark green and light green/white represent the highest and lowest number of proteins.

The genomic diversity and variability among the isolates are highlighted by the analysis of singletons and non-core genes that may be lost or acquired from distal lineages through horizontal gene transfer and represent the genetic source for the emergence of novel variants (Figure 2, Table S2). To assess the functional features of singletons, TIGRFAM was used to cluster proteins in categories and sub-categories (Figure 3). Genes involved in central metabolism as well as several hypothetical coding sequences and some with unknown functions are distributed over all the genomes. Moreover, a distinctive feature is the presence of prophage elements that are found only in MA-13, DSM1, and P38 (Figure 3). These strains are also characterized by singletons related to the functions of mobile genetic elements. This feature points to the availability of genes attainable by the evolving microbial population and to the capability of microbial genomes to acquire new functions. The distribution of singletons in the MA-13 strain revealed a significant proportion (~40%) of them falling within the hypothetical proteins category with unknown function thus indicating that a significant portion of them is still “terra incognita” and therefore the full understanding of its genomic potential needs to be still unveiled (Table S3). Moreover, transposable elements provide microorganisms with the ability to be responsive and susceptible to environmental changes by acquiring new genetic material and disseminating regulatory elements. The presence of singletons related to transposon activity, which is a distinctive feature of MA-13, points to significant genome plasticity, possibly to meet the requirements of environmental adaptation.

### 2.3. CAZome of W. coagulans

CAZymes repertoire covers diverse enzymatic functions related to the synthesis, modification, and breakdown of saccharides, thus exploiting a great potential in biotechnological applications. *W. coagulans* CAZome was explored through two different tools, i.e., dbCAN 2 metaserver and the app developed for KBase “Search with dbCAN2 HMMs of CAZy families” (Table 2).

The dbCAN tool has been extensively used over the last ten years to annotate CAZymes in genomes/metagenomes and compared to the CAZy website is suitable for comparative analysis [27,28]. Indeed, for instance only three out of the nine genomes were present in the CAZy database. Moreover, the searching parameters in this online database are not specified, thus accounting for differences in the total number of enzymes active on carbohydrates that can be found with other tools. For these reasons, we resolved to investigate on *W. coagulans* CAZome with the tool “Search with dbCAN2 HMMs of CAZy families” and the results were compared with those obtained with dbCAN 2 metaserver. The former allows a comparative search through the genes of the nine selected *W. coagulans* genomes by HMMER models according to the dbCAN CAZyme domain HMM database. Instead, the output of the latter are lists of CAZymes identified with three different tools databases (HMMER, DIAMOND, eCAMI), thus resulting in incongruencies among the analyses. Interestingly, by comparing the HMMER data obtained with the two methods, we observed differences in the total number of GTs, CEs, AAs, and CBMs (i.e., Glycosyl Transferases, Carboxyl Esterases, Auxiliary Activities and Carbohydrate Binding Modules, respectively) but not for GHs (Glycoside Hydrolases) (Figure S1). This bias can be traced back to the highest number of available structures/models for GH enzymes than for the other classes of CAZymes. Moreover, the number of GTs, CEs, AAs, and CBMs searched through dbCAN 2 metaserver over exceeds that identified by “Search with dbCAN2 HMMs of CAZy families” since the software parameters are not tunable in the metaserver.

**Figure 4:**
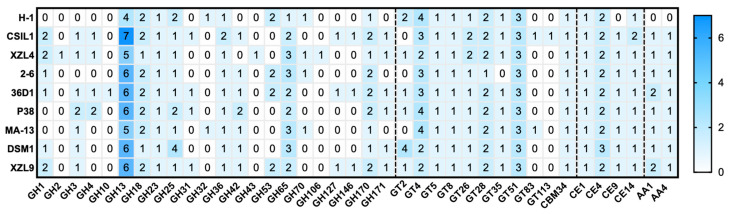
Heat map showing the genome-wide distribution of CAZymes in the *W. coagulans* strains. A total of 36 families were identified and obtained with Search with dbCAN2 HMMs of CAZy families. Blue and white represent the highest and lowest number of proteins.

The total amount of CAZymes in each of the nine genomes (393 genes in total) represents more than ~1% of all predicted coding sequences (Table 2). A deeper inspection of the CAZome is reported in Figure 4. The annotated genes were clustered in glycoside hydrolases (50.6%), glycosyltransferases (30.8%), carbohydrate esterases (11.7%), and auxiliary activity (4.6%) families.

Glycoside Hydrolases (GHs) catalyze the hydrolytic cleavage of the glycosidic bond and are involved in the degradation of agri-food and lignocellulose biomasses [29,30,31,32,33,34]. A total of 199 genes encoding for GHs are clustered in 22 families, among which some are spread over all the genomes, i.e., GH13, GH18, GH23, GH25, GH36, GH65, and GH170. The highest number of sequences (51 and 21, respectively) were found within GH13 and GH65 families whose members are mainly active on substrates containing α-glucoside linkages, such as starch [35]. GH42 representatives are found in almost all the strains except for H-1 and XZL4. Sequence analysis and experimental data indicate that the GH42 enzymes are mostly beta-galactosidases which are key providers of energy and carbons source through the breakdown of lactose to galactose and glucose [36]. GHs members belonging to families 18 might be involved in the spore germination and this observation is in line with the lifestyle of *W. coagulans* members. In particular, the GH10 member was retrieved in the singletons list of *W. coagulans* 36D1, suggesting that this enzyme was acquired through horizontal gene transfer among bacterial genomes.

Glycosyl Transferases (GTs) are enzymes that catalyze the transfer of sugar moieties from activated donors to specific acceptor molecules, forming glycosidic bonds, and are involved in the synthesis of oligosaccharides, polysaccharides, and glycoconjugates [37]. Typically, the bacterial glycosyltransferases are poly-specific enzymes that mainly belong to families GT2 and GT4 and exploit the inverting and retaining catalytic mechanism, respectively. Except for GT2, GT35, GT83, and GT113, at least one member of the remaining identified GTs is found in all the genomes. Moreover, GT4/GT 51 and GT83/GT113 are the largest and the smallest groups, respectively. Noteworthy, the GT83 family which includes enzymes involved in lipopolysaccharide biosynthesis [38], typifies the GT repertoire of only two strains, i.e., MA-13 and CSIL1. Finally, family GT28 related to peptidoglycan biosynthesis, maintenance of the rod cell shape, and elongation of the lateral cell, is generally represented by two members for each genome.

Carboxyl Esterases (CEs) release acyl or alkyl groups attached by an ester linkage to carbohydrates [39]. Among the 18 CEs families, only four of them were annotated in this analysis. CE1, CE4, CE9, and CE14 are found in almost all the strains.

The remaining enzymes are categorized as Auxiliary Activities (AAs) in the CAZy database and comprise carbohydrate oxidases, redox enzymes involved in diverse functions such as lignin degradation and metabolism of lignin-derived compounds [40]. AA members (in total 18) were assigned to AA1 and AA4 families; the former are multicopper oxidases that use diphenols and related substances as donors and oxygen as acceptors [41], while the latter are potential vanillyl-alcohol oxidases [42].

Finally, a single representative of CBM 34 family has been identified in all the genomes. Little is known about the function of CBM 34 members; indeed granular starch-binding function has been demonstrated only in the case of *Thermoactinomyces vulgaris* R-47 α-amylase 1 [43].

The CAZome analysis along with the available biochemical and physiological data in the literature points to *W. coagulans* as a model microorganism not only for carbohydrate degradation processes but also as a valuable source of novel enzymes for potential applications especially in nutraceutical, food, and medical industries. Specifically, MA-13 is currently tested for its ability to use agri-food wastes as carbon and energy sources (manuscript in preparation), showing excellent degradative capability on diverse biomasses. Since enzymatic activities related to cellulose degradation are missing, *W. coagulans* has the potential to be used in the isolation of cellulose from agri-food wastes.

### 2.4. Protein Secretion Systems

Bacterial cells rely on a plethora of secretion systems to dynamically interact with their environment. Two main types of protein secretion systems, the Sec and Tat pathways are the most common ones and have been identified in all domains of life [44]. Both Tat and Sec systems are present in W. coagulans as shown by the core set that includes tatC/tatA and secYEG genes [4]. Moreover, the comparative analysis of non-core genes revealed that W. coagulans exploits other less widespread secretion systems (Table 2). Indeed, 36D1 and CSL1 bear the hylD gene that is a component of the Escherichia coli α-hemolysin secretion system (type I secretion system) which is otherwise made up also of hlyA, hlyB, and tolC [45]. Most, but not all, W. coagulans strains are featured by genes of the type VII secretion system (cT7SS) which exports proteins lacking a canonical cleavable signal peptide but bearing a WXG motif at the N-terminus [46]. EsxA and EsxB are molecular markers of the T7SS system and the corresponding genes (esxA and esxB) are usually part of clusters each harboring coding sequences for a membrane-associated ATPase of the FtsK–SpoIIIE protein family and for other putative components of the secretion system. Only P38 bears a complete set of the main components, whereas all the others show only some of them (Table 3).

It can be hypothesized that either some of the T7SS genes are dispensable for leader-less secretion systems or that single components might cross-function with Tat or Sec systems [44,47]. For instance, MA-13 bears *essA*, *essB,* and *essC* which encode for transmembrane proteins, the latter with a demonstrated ATP-ase activity. It is worth noting the absence of *esxA* whose product usually work in association with EssA, EssB, and EssC. Moreover, we have demonstrated in a previous work that WXG-proteins not even related to virulence factors are secreted in MA-13 through a leader-less mechanism, suggesting that *esxA* is dispensable for this secretion system in this strain [15]. Interestingly, a homologue (38% identity) of *B. subtilis* EsxA has been found in some *W. coagulans* strains (36D1, 2–6 and P38) but there are no reports about its functional characterization in *W. coagulans*. In biotechnological applications, it would be useful to facilitate protein production or protein downstream processing using different secretion mechanisms for biotransformations and/or for screening libraries of enzyme variants. The study of the multiple secretion systems functioning in MA-13 along with its fermentation performance [7,8] makes this host a suitable starting point for the development of a consolidated bioprocess for the production of recombinant proteins/enzymes of biotechnological interest.

### 2.5. Resistance Mechanisms to Environmental Challenges

#### 2.5.1. Toxin–Antitoxin Systems

Toxin–antitoxin (TA) systems are ubiquitous among bacteria and play a crucial role in the dissemination and evolution of antibiotic resistance, for instance through stabilization of multi-resistant mobile genetic elements and phages [48,49]. Generally, toxins are proteins that affect metabolism and antitoxins are either RNA or proteins that counteract the effect of the toxin. Currently, TA systems are based on the coordinated action of a toxin which is always a stable protein, while the labile antitoxins can be either RNAs or proteins [50].

All *W. coagulans* strains are endowed with a common TA system which relies on *ydcD mazF* and *ydcE/mazF* genes, encoding for both an endoribonuclease that inactivates cellular mRNAs and its inhibitor, respectively. Moreover, we spotlighted in the MA-13 singletons set, the presence of a gene encoding for sequence-specific endoribonuclease belonging to PemK/MazF family (MBF8417901.1) [51] and lying upstream of an ORF encoding for a putative antitoxin of the AbrB/MazE/SpoVT family (MBF8417900.1). The TA systems of *W. coagulans* are a platform to discover novel antimicrobial targets, as well as to set up genetic reporter systems for plasmid maintenance and protein production [52].

#### 2.5.2. Genetic Determinants of Resistance to Bacitracin

**Figure 5:**
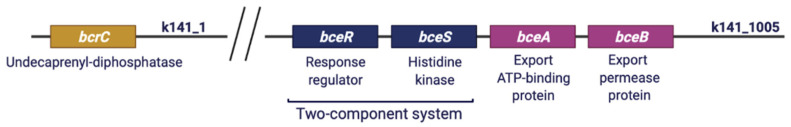
Genetic determinants of bacitracin resistance in MA-13.

Another strategy to withstand exposure to toxic compounds is antimicrobial resistance (AMR) which refers to the ability of microorganisms to cope with the effects of antibiotic treatments or antimicrobial peptides [53]. To identify genomic regions possibly related to AMR, an in silico analysis of *W. coagulans* “resistome” was performed using PATRIC software. The output of this analysis is a list of gene products that are mostly related to functions of the central metabolism and are potential targets of environmental stressors upon genetic mutations. Then, PATRIC results need careful interpretation since the presence or the absence of some genes cannot be traced back to susceptibility or resistance to certain compounds unless biochemical and/or physiological characterization are carried out. Examples are *gyrA*, *gyrB*, and *murA* present in all the strains that might be antimicrobial target of fluoroquinolone (Table S4). However, no inference can be drawn in regard of fluroquinolone resistance/susceptibility since a physiological characterization has not been carried out for any of the strains. Interestingly, PATRIC analysis revealed the presence of a gene cluster encoding for resistance to a cyclic peptide (bacitracin) in MA-13. In antibiotic-producing bacteria, it is known that biosynthetic operons of peptide antibiotics are frequently associated with membrane drug efflux transporters of the ABC family, which pump out the antibiotics for self-protection [54,55]. Accordingly, all *W. coagulans* strains analyzed bear three genes encoding for efflux pumps (*bceA*, *bceB*, *bcrC*). Furthermore, MA-13 is the only strain with a complete genetic array responsible for resistance to bacitracin including the two-component signal transduction system (*bceRS*) which is adjacent to the efflux transporter genes (*bceA/B*) (Figure 5). This genetic proximity is common to other *Bacillus* species [55] and the relative genes are located on contig k141_1005 in MA-13. In this system, BceR is a sensory transduction protein that responds to extracellular bacitracin stimulus and transmits the signal to the regulator BceS. This latter binds the promoter region upstream to *bceA/B* genes affecting their expression. Whilst the presence of a complete *bceRS/AB* gene cluster is quite widespread among other *Bacillus* species [55], MA-13 exhibits another genetic determinant of bacitracin resistance, i.e., *bcrC* encoding for a broad-specificity multidrug efflux pump which is located on a genomic region (contig k141_1) distant from the *bceRS/AB* genetic array. MA-13 BcrC protein is homologous (31.3% a.a. sequence identity) to BcrC of *Bacillus licheniformis*, a hydrophobic protein functioning as a membrane component of the permease [56]. Although some *Bacillus* species bear a complete three-components operon *bcrABC*, it has been demonstrated that the presence of the *bcrC* gene is sufficient to confer bacitracin resistance [57] (Figure 5). To sum up, the current analysis demonstrates the presence of putative gene target(s) that may account for tolerance to bacitracin.

Bacteria exposed to continuously changing chemico-physical conditions are expected to be endowed with features of better adaptability and competitive fitness to overcome environmental challenges. The horizontal gene transfer (conjugation, transformation, and transduction) and the role played by mobile genetic elements (plasmids, transposons, insertion sequences, integrons, and integrative-conjugative elements) and the bacterial toxin–antitoxin system as well as the occurrence of genetic mutations, lead to a speedy bloom of antibiotic resistance amongst bacteria [22].

#### 2.5.3. Secondary Metabolites: Bacteriocins

The discovery of new natural compounds produced by microorganisms, the so-called secondary metabolites, is a hot topic for diverse biotechnological applications [58]. These bioactive molecules, such as pigments, alkaloids, toxins, and antimicrobials, are not involved in the basal metabolism, rather they often perform ancillary functions [59]. The ability of *W. coagulans* to hinder bacterial growth and balance co-habitant microbiota populations by producing antimicrobial compounds, was already demonstrated [16].

**Figure 6:**
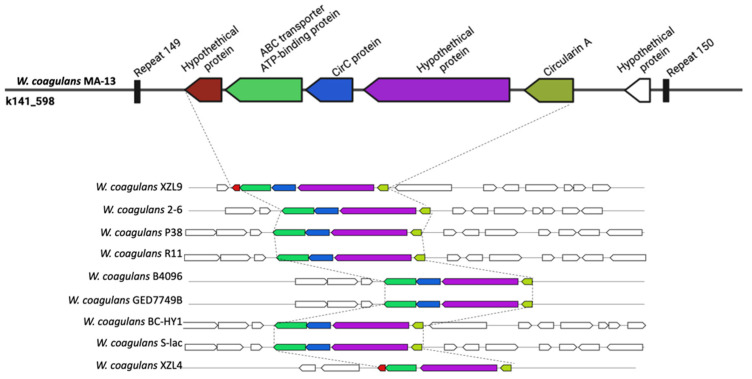
Graphical representation of circularin A gene cluster in diverse *W. coagulans* strains.

To understand the potential of MA-13 in the production of secondary metabolites, the genome was analyzed using antiSMASH, a bioinformatic tool suitable to search for clusters involved in secondary metabolites synthesis (Figure 6). From this analysis, a gene encoding for a non-lanthionine-containing peptide (class II bacteriocins), the circularin A, was identified. Circularin A is usually produced as a pre-peptide that undergoes a proteolytic cleavage of the leader sequence followed by head-to-tail ligation between the N- and C-termini to produce a smaller circular antimicrobial peptide [60]. This bacteriocin is usually encoded by a set of 4–10 genes usually organized in cluster. A genetic arrangement formed by ORFs encoding for circularin A, CircC, ABC transporter, and two hypothetical proteins were found in MA-13 on contig k141_585. A similar structure is shared with almost all the strains analyzed (Figure 6), but in the case of MA-13, the region is delimited by two repeats. Moreover, given the presence of several surrounding mobile elements, among which a transposase IS4, it is likely that the acquisition of this genetic cassette occurred upon HGT in MA-13. The strain XZL4 is the only one missing the *circC* gene, whose product is known to have a central role in the maturation of circularin A. Bacteriocin production could be considered advantageous to the producer since these peptides can kill or inhibit bacteria by competing for the same ecological niche or the same nutrient pool. Noteworthy, probiotics that synthetize bacteriocins are of particular importance because these bacteria are widely used in dairy industries.

### 2.6. Innate and Adaptive Immunity

#### 2.6.1. Innate Immunity

**Figure 7:**
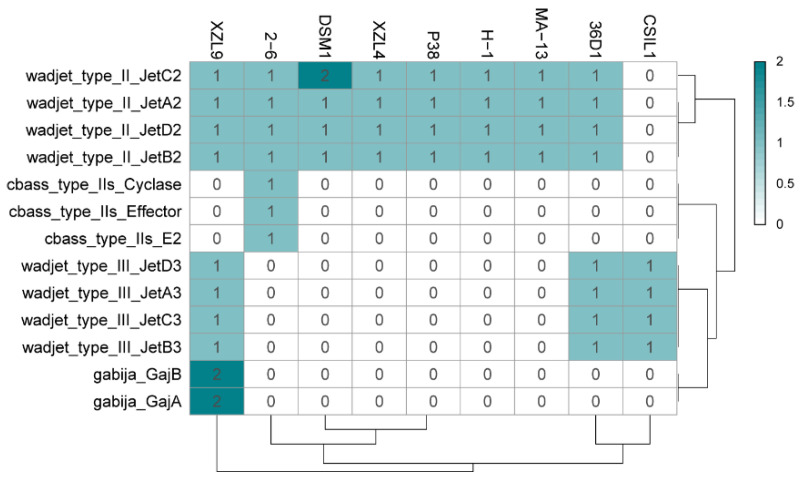
Heat map distribution of innate immunity systems in the *W. coagulans* strains. Green and white represent the highest and lowest number of proteins.

Prokaryotic immunity is constituted by multiple systems to defend from mobile genetic elements (i.e., viruses and plasmids). Innate defense mechanisms do not require previous exposure to a pathogen. The examination of the different *W. coagulans* genomes revealed the ubiquitous presence of the Wadjet defence immune system in all the strains analyzed (Figure 7 and Table S5) [61]. Wadjet cassettes are composed of the four genes *jetABCD*, where JetABC are homologs of the bacterial condensins MukF, MukE, and MukB, respectively. Bacterial condensins are involved in DNA condensation and segregation during bacterial replication. The fourth component of the operon, JetD, has a putative topoisomerase VI domain. Three types of Wadjet systems have been described, reflecting the domain organization of its components within the cassette [62]. *W. coagulans* strains predominantly contain type II Wadjet systems, although 36D1, CSIL1, and XZL9 harbor type III Wadjet operons (Figure 7). Wadjet system has been related to antiplasmid defense in the Gram-positives *Bacillus cereus* Q1 and *Mycobacterium smegmatis* [63] where it impairs plasmid transformation and plasmid segregation to daughter cells, respectively. It can be hypothesized that the presence of Wadjet systems in *W. coagulans* is involved in preventing the acquisition of DNA from the environment. This would hamper the development of genetic systems for these strains, similarly to what occurs in *M. smegmatis,* where disruption of this system allowed the transformation and maintenance of plasmid pAL5000 [63]. Other isolated defense systems are present in *W. coagulans*. Strains 2–6 harbor a single CBASS (cyclic-oligonucleotide-based anti-phage signaling systems) cassette in addition to a type II Wadjet system (Figure 7) [64]. CBASS systems are constituted by an oligonucleotide cyclase that produces cyclic nucleotide secondary messengers upon the encounter of a pathogen, and an effector protein. They are classified into four types according to their operon organization, type of effector, and cyclic nucleotide messenger. In particular, effectors of type II CBASS systems contain ubiquitin-associated domains and their mechanism of action are still unknown given that ubiquitin has not been found in bacteria. However, this system confers protection against phages in *E. coli* and *V. cholerae.* Strain XZL9 harbors two Wadjet cassettes of types II and III, plus two Gabija loci (Figure 7). The Gabija system is composed of proteins GajA and GajB where GajA is a sequence-specific DNA nicking endonuclease and GajB is predicted to be a UvrD-like helicase. It is that GajA nuclease activity is activated by the change in the intracellular nucleotide levels resulting from virus replication and transcription, thus eliciting bacteriophage resistance [65].

Similar to many environmental strains, *W. coagulans* is not amenable to transformation with exogenous DNA and so far only two strains (DSM1 and P4-102B) are genetically tractable. However, setting up a reproducible genetic system is indispensable to apply metabolic engineering techniques to meet the requirements of commercial applications. The ubiquitous presence of the putative antiplasmid Wadjet immune system in *W. coagulans* may explain the recalcitrance of these strains to genetic tractability and the deletion of this anti-plasmid cassette constitutes a promising strategy for the development of strains amenable to genetic engineering.

#### 2.6.2. Adaptive Immunity: CRISPR-Cas System and Mobile Elements

CRISPR-Cas systems constitute an adaptive immune apparatus in a wide range of bacteria (~40%), and the majority of archaea (~90%); small guide RNAs (crRNAs) interfere with sequence-specific nucleic acids neutralizing invading genetic elements [66]. A CRISPR locus consists of repeats (23–47 bp) separated by unique sequences (spacers) with similar length (21–72 bp), originating from mobile genetic elements such as bacteriophages, transposons, or plasmids. Moreover, the *cas* genes are generally closely linked to CRISPR arrays and are diversified. Indeed, CRISPR-Cas systems are classified into two classes which are further subclassified into six types and several sub-types: in class I, the effector module consists of several Cas proteins whereas class II is defined by the presence of a single multidomain protein (e.g., Cas9). The CRISPR-Cas defense mechanism acts in a sequence-specific manner by recognizing and cleaving foreign DNA or RNA. The defense response can be divided into three stages: (i) adaptation or spacer acquisition, (ii) crRNA biogenesis, and (iii) target interference [67].

**Figure 8:**
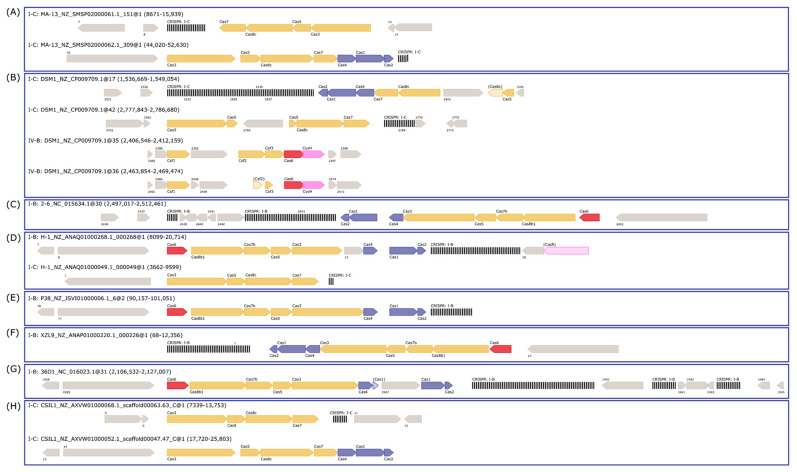
CRISPR-Cas cassettes present in *W. coagulans* genomes. The *cas* operons for each strain are depicted: (**A**) MA-13, (**B**) DSM1, (**C**) 2–6, (**D**) H-1, (**E**) P38, (**F**) XZL9, (**G**) 36D1, and (**H**) CSIL1. The headers above each operon refer to the type of *cas* operon, strain, contig, and location. The color of *cas* genes corresponds to their associated stage of the CRISPR-Cas response: blue—adaptation; yellow—interference; and red—crRNA maturation. CRISPR arrays are depicted as black, vertical lines.

**Figure 9:**
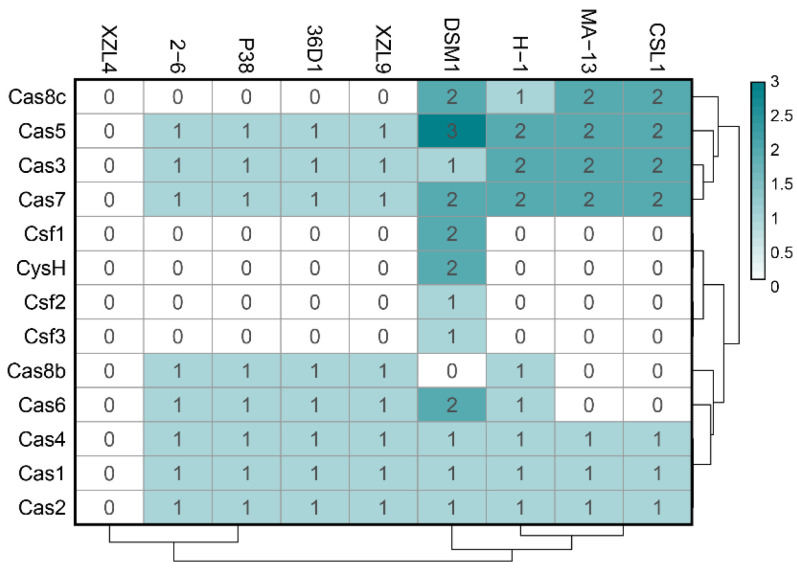
Heat map distribution of *cas* operon genes among *W. coagulans* strains. Green and white represent the highest and lowest number of genes.

MA-13 genome harbors 48 spacers, 51 repeats organized in at least 2CRISPR arrays with the same direct repeat sequence. The exact number of arrays cannot be determined due to the fragmented nature of the MA-13 genomic assembly that includes small contigs containing partial, truncated CRISPR arrays. Additionally, these arrays are linked to several unidirectionally arranged *cas* genes organized in two loci (*cas1*, *cas2*, *cas3*, *cas4*, *cas5*, *cas7*, and *cas8*) (Figure 8 and Table S6). Such genomic association is typical of Class I-C, characterized by the lack of *cas6*, encoding for an endoribonuclease employed by most type I systems for pre-crRNA processing and by the presence of a single protein encoded by the *cas8c* gene. The absence of *cas6* can be replaced by *cas5* whose product performs a similar catalytic reaction. In type I-C system, genes are typically encoded by a single (predicted) operon. Genes *cas 3*, *5*, *8c*, and *7* of locus 1 share sequence identity below the 30% threshold with the corresponding genes located on the other locus. This indicates that these *cas* genes do not derive from gene duplication rather they have been acquired through independent events (see below). The comparative genomic analysis revealed that eight strains of *W. coagulans* possess *cas* operons of type I-B (2–6, 36D1, H-1, P-38, and XZL9), type I-C (CSIL1, DSM1, H-1, MA-13), or type IV (DSM1), mostly located in the vicinity of CRISPR arrays (Figure 8 and Figure 9 and Table 4). Both type IV *cas* operons are located within prophages in the DSM1 genome, suggesting that they are of viral origin. Only strain XZL4 lacks a complete *cas* operon or CRISPR array.

The *W. coagulans* strains contain 1 to 5 CRISPR arrays each, for a total of 30 arrays of which 13 are annotated as orphan CRISPRs (i. e. not located in the vicinity of a *cas* operon). Additionally, while strains MA-13, P38, and XZL9 have one consensus direct repeat sequence, strains 36D1, DSM1, CSIL1, and H1 have two to three direct repeat sequences despite all strains harboring one single adaptation cassette (Table 4).

**Figure 10:**
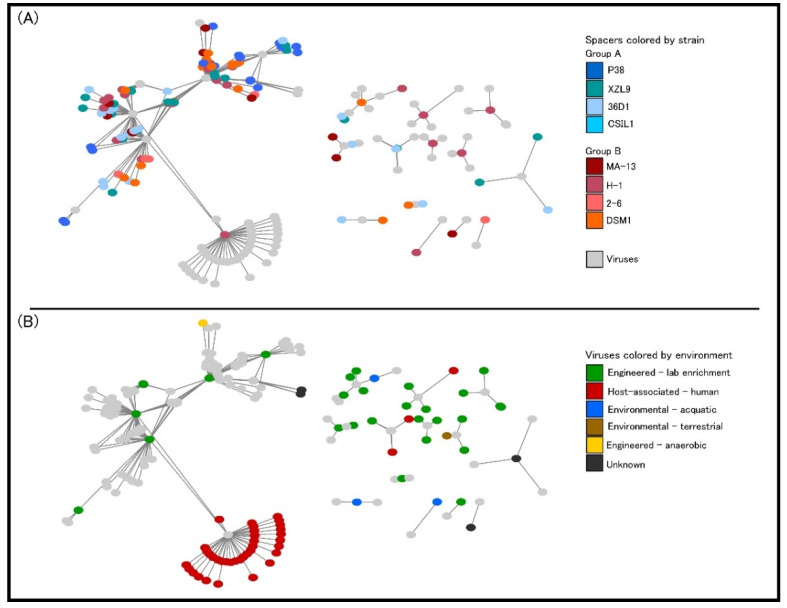
Graphical representation of interaction networks between spacers and viruses. Nodes represent spacers and virus sequences, with lines representing a match between them. (**A**) Spacer nodes colored by their strain of origin. (**B**) Virus nodes are colored by their ecosystem of isolation.

At present, it is not clear whether orphan CRISPR arrays are indeed functional, although several lines of evidence indicate that at least some isolated CRISPR arrays are active [68,69]. Strains 2–6, 36D1, and CSIL1 have orphan arrays with a repeat different to the one of the CRISPR array associated with their *cas* operon and adaptation cassette. Moreover, in strains 36D1 and CSIL1 this repeat is predicted to belong to another CRISPR subtype. A similar picture is seen for strain DSM1, which has an adaptation cassette associated with an I-C CRISPR system, but an orphan array with a different direct repeat. These arrays could represent a remnant of a *cas* operon previously present in the genome that was subsequently lost. Additionally, strain H-1 bears interference cassettes for both I-B and I-C subtypes, but an adaptation cassette only for the I-B operon (Table 4). The repeat of the associated CRISPR arrays is different for each subtype, suggesting that both *cas* operons are capable of interference, but only the I-B cassette may be able to acquire new spacers. The low number of spacers in the I-C CRISPR arrays (3 to 5) could reflect its inability to acquire new spacers. Taken together, the distribution of CRISPR-Cas modules in *W. coagulans* strains suggests that loss and gain of CRISPR-Cas cassettes is a common trait in these microorganisms. From an applicative point of view, the endogenous CRISPR-Cas adaptive immune systems can be repurposed for genome editing of *W. coagulans,* potentially constituting a valuable tool in the genetic toolbox for these strains. Analysis of CRISPR spacer matches allowed the identification of viruses infecting the *W. coagulans*. From a pool of 651 spacers extracted from the strains, 113 spacers (17.3%) matched to 77 *Firmicutes-*infecting viruses in the IMG-VR database [70]. These viruses are classified as members of the class *Caudoviricetes,* with the majority of them (66%) belonging to the *Siphoviridae* family. Several viruses match to CRISPR spacers of strains from both clades of *W. coagulans* (group A and group B), suggesting that these strains share a common pool of viruses. Additionally, most viruses in the network originate from an engineered environment (e.g., lab enrichment), including all spacers from strain MA-13 (Figure 10B), while only four viruses derived from environmental ecosystems. Interestingly, one single spacer from strain H-1 matched 38 viruses, of which 36 originate from host-associated environments, particularly the human digestive system (Figure 10). The source of the *W. coagulans* viruses is in agreement with the prevalent agri-food origin of the isolated *W. coagulans* strains.

**Figure 11:**
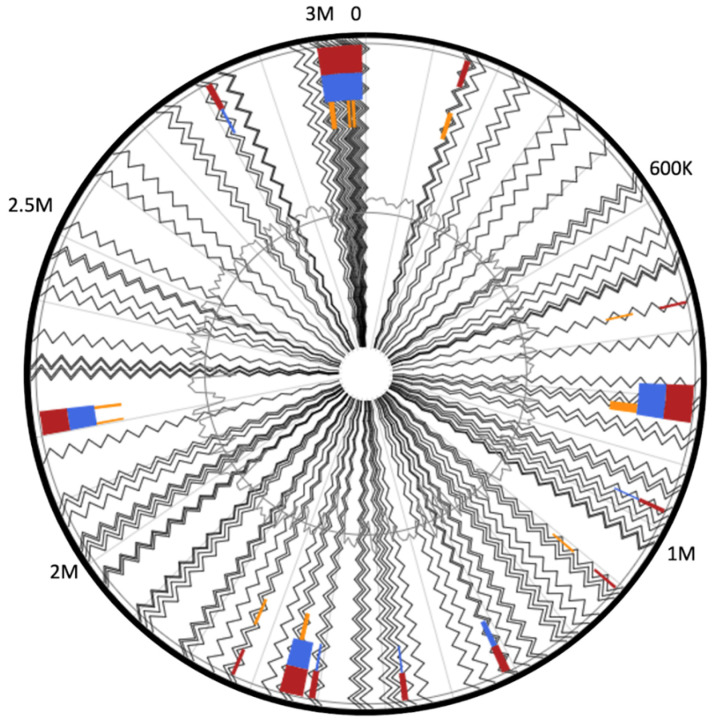
Circular visualization of predicted genomic islands. Blocks are colored according to the prediction method: IslandPath-DIMOB (blue), SIGI-HMM (orange), as well as the integrated results (dark red).

The analysis of the adaptive immunity in MA-13 was extended also to the identification of genomic islands (GIs, regions of probable horizontal origin) and prophage regions [71,72]. GIs are non-self-mobilizing integrative regions encoding factors that support the adaptability and competitiveness of the microbes within a niche, including virulence factors and other medically or environmentally important adaptations. Using IslandViewer, the prediction of GIs is carried out with a precise definition of boundaries since integration splits off a gene fragment that marks the distal ends of the island. In total, 297 genes were assigned as belonging to GIs (Table S8 and Figure 10), among which the following *cas* genes have been identified: *cas3* (MBF8417476.1), *cas5* (MBF8417475.1), *cas8c* (MBF8417474.1), *cas7* (MBF8417473.1, MBF8417526.1), *cas4* (MBF8417527.1), *cas1* (MBF8417528.1), and *cas2* (MBF8417529.1). A similar analysis conducted on strains 2–6 and 36D1 indicates that the CRISPRs-Cas systems are located in genomic islands that are flanked by transposases [4]. Several CRISPR-Cas systems have been discovered within GIs and for *V. cholerae* all CRISPR-Cas arrays are located in mobile genetic elements (MGEs) [71]. Among the other genetic traits found in GIs that enhance the fitness of MA-13, worth noting is the presence of (i) two genes encoding for putative thiazole-containing heterocyclic bacteriocins (MBF8418016.1 bacteriocin biosynthesis cyclodehydratase) and uberolysin/carnocyclin family circular bacteriocin (MBF8417079.1); (ii) a full set of genes belonging to type I restriction systems that are large pentameric proteins with separate restriction (R, MBF8419113.1), methylation (M, MBF8419111.1), and DNA sequence-recognition (S, MBF8419112.1) subunits; and (iii) a complete cluster of genes encoding for arsenic resistance made up of *arsABCD* (MBF8417195.1, MBF8417192.1, MBF8417193.1, MBF8417194.1). ArsB and ArsD are arsenite efflux transporters, ArsC is an arsenate reductase and ArsA is an arsenical pump-driving ATPase that overall contributes to confer resistance to arsenic (Figure 11, Table S8) [73].

**Figure 12:**
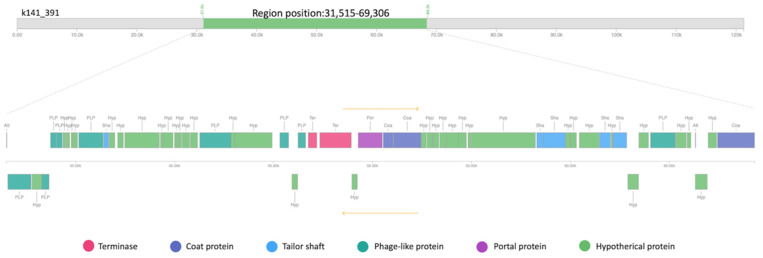
Linear genomic map of prophage phBC6A52 identified with PHASTER from MA-13 genome.

Furthermore, the prophage finding software PHASTER was used to identify intact prophages and their integrase genes in MA-13, utilizing a BLASTP comparison of the query genome with a frequently updated prophage sequence database [74]. Only one prophage is present in the MA-13 genome whose integrity is indicated by the presence of the attachment junctions *attL* and *attR* (also named hybrid attachment sites) at the extremities of the prophage (Figure 11). A total of 58 genes were identified in this genomic region that overall represent a blend of bacterial and bacteriophage features. Specifically, 20 hypothetical proteins were annotated using Bacterial Database and the remaining 38 belong to diverse phages (Figure 12 and Table S9). Among these genes, 13 are linked to *Bacillus cereus* prophage phBC6A52 (Genbank NC_004821.1) assigned to the *Podoviridae* family [75]. Besides MA-13, P38 also bears a complete sequence related to a *B. cereus* prophage. PHASTER analysis revealed that it shares 100% identity with *Bacillus* Phage vB_BtS_B83 that belongs to the *Bembunaquatrovirus* bacteriophages genus. Although bacteriophages typically exhibit a narrow host range, yet the presence of the same phage in two different *Bacillus* species suggests that the horizontal gene transfer events can overcome the interspecies boundaries to allow the bacteria–phage coevolution in a given ecotype. Indeed, the exchange of phage attachment molecules could even occur in an interspecies context, enabling phage adsorption to non-host species, providing an alternative route for HGT. In in vitro models, species of *Bacillus*, including *W. coagulans, B. subtilis,* and *B. cereus* showed a similar life cycle in artificial gastrointestinal tract models [76].

Since *W. coagulans* and *B. cereus* strains have both been isolated mainly from food sources, it is reasonable that they share the same lifestyle and/or ecological environment, making it possible for genetic material exchanges [77,78].

## 3. Materials and Methods

### 3.1. Genome Assembly and Annotation

MA-13 genomic sequence was re-assembled using MEGAHIT v 1.2.9 [79]. The result was analyzed using QUAST v 4.4 [80] and the assembly quality was evaluated using CheckM v 1.0.18 [81]. PATRIC (i.e., NIAID/PathoSystems Resource Integration Center) was used for resistome analysis as well as for comparative genomic analysis [53]. AntiSmash v 2.0 (https://antismash.secondarymetabolites.org, accessed on 16 July 2021) was employed to analyze secondary metabolite biosynthesis gene clusters [82].

### 3.2. Construction of Whole-Genomes Sequence-Based Phylogenetic Tree and Comparative Analysis of Orthologous Genes

The genomes of 47 *W. coagulans* strains available at the NCBI (on 1 February 2022) (https://www.ncbi.nlm.nih.gov/genome) were imported into KBase system (on 14 June 2021) to create a genome set, that subsequently was employed to build the tree, using SpeciesTreeBuilder v 0.1 [83]. Afterward, the genomic comparison was performed using Build Pangenome with OrthoMCL (Pangenome Orthomcl v 0.0.7) and visualized with Pangenome Circle Plot app (v 1.2.0) [26]. The whole-genome sequences of the 9 strains, namely *W. coagulans* MA-13 (SMSP00000000), *W. coagulans* 2–6 (NC_015634), *W. coagulans* 36D1 (NC_016023), *W. coagulans* CSIL1 (NZ_AXVW01000001), *W. coagulans* DSM1 (NZ_CP009709), *W. coagulans* H-1 (NZ_ANAQ01000001), *W. coagulans* P38 (NZ_JSVI01000001), *W. coagulans* XZL4 (NZ_AFWM01000001), and *W. coagulans* XZL9 (NZ_ANAP01000008) were included to evaluate the clusters of orthologous groups (COGs) and to identify the core, non-core, and singletons. These last ones were also compared by annotating the functional domains in the whole genome set with DomainAnnotation (version 1.03) using TIGRFAM [84] and viewing the results using the View Function Profile for Genomes app (v 1.0.1). Furthermore, the CAZome was annotated using dbCAN 2 Meta Server (https://bcb.unl.edu/dbCAN2/index.php, accessed on 10 February 2022) and “Search with dbCAN HMMs of CAZy families” (v 10.0; urly.it/3hhmd) in KBase, using an e-value of 1 × e^−15^, a bit score 100 and an alignment overlap threshold of 35% [27,83].

Two strains, i.e., MA-13 and DSM1 were selected based on their genetic relatedness and by pairwise comparative analysis by calculating the average nucleotide identity (ANI) using FastANI. The percentage threshold for species boundary is 95% ANI [85]. In silico DNA–DNA hybridization (DDH) values were calculated using the Genome-to-Genome Distance Calculator (GGDC) v 3.0 [24].

### 3.3. Analysis of Resistance Mechanisms to Environmental Challenges

Identification of prokaryotic innate defense systems in the *W. coagulans* genomes was carried out using the webtool PADLOC (https://padloc.otago.ac.nz/padloc/, accessed on 16 September 2021) [86]. CRISPR/Cas systems were identified with the web tool CRISPRCasTyper (https://crisprcastyper.crispr.dk/, accessed on 15 September 2021) [87] and the orientation of the CRISPR arrays was determined using CRISPRDetect (http://crispr.otago.ac.nz/CRISPRDetect/predict_crispr_array.html, accessed on 15 September 2021). CRISPR spacers predicted by CRISPRCasTyper were compared to the 2,332,702 sequences in the IMG-VR database (downloaded on the 22 August 2021) [70] using BLAST (blastn-short task, e-value of 1 × 10^−5^). The resulting network of the virus–host interactions was visualized using Cytoscape (https://cytoscape.org/v.3.7.0, downloaded on 4 December 2018). The IslandViewer4 web server (http://www.pathogenomics.sfu.ca/islandviewer/, accessed on 4 December 2018) was utilized for the prediction and interactive visualization of genomic islands (GIs) whose validation has to be performed with at least one of the softwares (IslandPick, SIGI-HMM, or IslandPath-DIMOB) [88]. The web server PHASTER (http://phaster.ca, accessed on 10 October 2021) uses BLAST against a phage-specific sequence database to predict prophage(s) integrated into the bacterial genomes [74]. Only prophages identified as “complete” or “intact” are considered.

## 4. Conclusions

Thermophilic microorganisms are a reservoir for biodiversity, molecular phylogeny, and the production of unique industrially valuable enzymes and other compounds [89,90,91,92,93,94,95,96,97]. The purpose of this work is to expand our understanding of the intra-strain genomic diversity of *W. coagulans* and to provide new insights into its genetic potential in biotechnological applications. Moreover, our analysis also contributes to the exploration of genomic diversity and sheds light on genetic traits that are essential to the basic lifestyle of *W. coagulans*. Furthermore, we have expanded the knowledge of the selective advantages that shape the organization and dynamics of *W. coagulans* genomes, including niche adaptation, antibiotic resistance, and genetic tractability.

It is noteworthy to underline that this work provides a comparative analysis among the nine strains based on annotation on NCBI and/or the specific bioinformatics tools used. The heterogeneity of how data are analyzed, annotated, and displayed and sometimes the lack of connectivity among the available data represent the most frequent issues related to bioinformatics studies that need wet-lab experiments to be validated.

The analysis of CAZymes did not highlight significant strain-specific differences. Moreover, the amount of CAZymes represents about 1–1.5% of coding sequences for all the strains tested. GH13 genes, that are related to starch degradation, are particularly abundant. However, the lack of information regarding the isolation source of these strains, leaves this matter murky. The comparative study of secretion systems sheds light on the presence of Tat and Sec genes in all the genomes and some sporadic components belonging to type I and VII systems have been identified in some of the strains. Although experimental data on protein secretion are currently available only for MA-13, the presence of diverse pathways to export proteins makes *W. coagulans* an excellent strain for biotransformation and homologous/heterologous protein expression. The nine strains analyzed have the potential to face environmental challenges through diverse resistance mechanisms. Differences among these strategies are likely related to the environment in which microorganisms thrive or have developed.

Analysis of immune systems involved in the defense against mobile genetic elements revealed the ubiquitous presence of the antiplasmid Wadjet system in the genomes analyzed, which could explain the refractory response of *W. coagulans* to genetic manipulation. Regarding adaptive immunity, although type I-B and type I-C CRISPR-Cas systems are widespread in the analyzed genomes, their patchy distribution and the presence of incomplete Cas protein cassettes and stand-alone CRISPR arrays imply that the gain and loss of CRISPR-Cas systems is a frequent trait in *W. coagulans*. This underlines the existence of a constant arms race with viruses supported by the CRISPR spacer matches and prophage prediction analyses. This aspect reveals a common pool of viruses of the *Caudoviricetes* infecting the different strains of *W. coagulans*.

So far, MA-13 is the strain which has received the most extensive physiological and biochemical characterization and therefore can be considered a model system for some interesting biotechnological applications. Indeed, the high operating temperature used during fermentation and its innate and adaptive immunity could protect the MA-13 strain from phage infection and contamination. The next step will be setting up a genetic toolbox for genome editing and metabolic engineering.

## Figures and Tables

**Table 1:** ANI value comparisons between the nine strains analyzed.

Query	Reference	ANI Estimate (%)
MA-13	CSL1	94.68
MA-13	36D1	94.85
MA-13	XZL9	95.04
MA-13	P38	95.15
MA-13	H-1	97.89
MA-13	2–6	97.98
MA-13	XZL4	98.22
MA-13	DSM1	98.43

**Table 2:** CAZymes distribution in the genomes of *W. coagulans* strains. The total number of genes (for each genome) is based on the data obtained by NCBI and the number of CAZymes has been derived from Search with dbCAN2 HMMs of CAZy families.

Strain	Total No. of Genes	No. of CAZymes	% CAZymes
MA-13	2689	40	1.49
DSM1	2766	45	1.62
36D1	3128	48	1.53
XZL9	3112	46	1.47
P38	3097	46	1.48
2–6	2743	39	1.42
H-1	2536	36	1.42
XZL4	2593	43	1.65
CSIL1	3165	50	1.56

**Table 3:** Non-core genes of *W.coagulans* secretion systems.

Accession Number	*W. coagulans* Strain	Function
WP_035181994.1	2–6	6 kDa early secretory antigenic target ESAT-6 (EsxA)
WP_041818879.1	2–6	Putative secretion accessory protein EsaA/YueB @ Bacteriophage SPP1 receptor
WP_013858856.1	2–6	FtsK/SpoIIIE family protein, putative EssC/YukB component of Type VII secretion system
WP_035181994.1	36D1	6 kDa early secretory antigenic target ESAT-6 (EsxA)
WP_014096028.1	36D1	secretion protein HlyD
WP_029141776.1	DSM_1	Putative secretion system component EssB/YukC
MBF8418457.1	MA-13	type VII secretion protein EssA
MBF8418459.1	MA-13	type VII secretion protein EssB
WP_026685044.1	CSIL1	secretion protein HlyD
WP_052123334.1	P38	FtsK/SpoIIIE family protein, putative EssC/YukB component of Type VII secretion system
WP_035190278.1	P38	FtsK/SpoIIIE family protein, putative EssC/YukB component of Type VII secretion system
WP_035190280.1	P38	Putative secretion system component EssB/YukC
WP_035190310.1	P38	Putative secretion accessory protein EsaA/YueB @ Bacteriophage SPP1 receptor
WP_035190312.1	P38	6 kDa early secretory antigenic target ESAT-6 (EsxA)

**Table 4:** CRISPR arrays in the *W. coagulans* strains.

Strain	Contig	Prediction	Start	End	Consensus_repeat	Orientation	Cas_associated	N_repeats
2–6	2–6_NC_015634.1	I-B	2,499,794	2,503,038	GTTGAACTTTAACATTGGATGTATTTAAAT	R	yes	50
		I-B	2,497,017	2,497,373	GTTTCAATTCCTCATAGGTAAAATACAAAC	R	yes	6
36D1	36D1_NC_016023.1	Unknown	320,637	321,284	TTTTGAAGCCGTCAAAAGGACAAAA	F	orphan	13
		I-B	1,096,065	1,097,943	GTTAGTATTTTACCTATGAGGAATTGAAAC	R	orphan	29
		I-B	2,117,433	2,121,795	GTTTGTATTTTACCTATGAGGAATTGAAAC	F	yes	66
		I-B	2,123,872	2,124,703	GTTTGTATTTTACCTATGAGGAATTGAAAC	F	yes	13
		I-B	2,126,172	2,127,007	GTTTGTATTTTACCTATGAGGAATTGAAAC	F	yes	13
CSIL1	CSIL1_NZ_AXVW01000068.1_scaffold00063.63_C	I-C	13,264	13,753	GTCACACTCCTTGCGAGTGTGTGGATTGAAAT	F	yes	8
	CSIL1_NZ_AXVW01000098.1_scaffold00091.91_C	I-C	7	236	GTCGCTCCCTACATGGGGGCGTGGATTGAAATC	F	orphan	4
	CSIL1_NZ_KI519465.1_scaffold00026.26	I-B	19,196	19,356	GTTAGTATTTTACCTATGAGGAATTGAAA	F	orphan	3
DSM1	DSM1_NZ_CP009709.1	I-C	1,536,669	1,541,901	GTCGCTCCCTACGTGGGGGCGTGGATTGAAAT	R	yes	80
		I-B	2,584,186	2,585,866	GTTAGTATTTTACCTATGAGGAATTGAAAC	F	orphan	26
		I-C	2,785,593	2,786,680	GTCACACTCCTCGTGAGTGTGTGGATTGAAAT	F	yes	17
H-1	H-1_NZ_ANAQ01000049.1_000049	I-C	9436	9599	GTCACACTCCTCGTGAGTGTGTGAATTGAAAT	F	yes	3
	H-1_NZ_ANAQ01000116.1_000116	I-C	43	204	GTCACACTCCTCGTGAGTGTGTGAATTGAAAT	F	orphan	3
	H-1_NZ_ANAQ01000125.1_000125	I-C	3689	3983	GTCACACTCCTCGTGAGTGTGTGAATTGAAAT	R	orphan	5
	H-1_NZ_ANAQ01000203.1_000203	I-B	74	2139	GTTGAACTTTAACATTGGATGTATTTAAAT	F	orphan	32
	H-1_NZ_ANAQ01000268.1_000268	I-B	17,520	20,714	GTTGAACTTTAACATTGGATGTATTTAAAT	F	yes	49
	H-1_NZ_ANAQ01000293.1_000293	I-B	112	339	GTTGAACTTTAACATTGGATGTATTTAAATT	R	orphan	4
MA-13	MA-13_NZ_SMSP02000017.1_69	I-C	32	1577	GTCGCTCCCTACATGGGGGCGTGGATTGAAAT	F	orphan	24
	MA-13_NZ_SMSP02000061.1_151	I-C	8671	10,021	GTCGCTCCCTACATGGGGGCGTGGATTGAAAT	R	yes	21
	MA-13_NZ_SMSP02000062.1_309	I-C	52,270	52,630	GTCGCTCCCTACATGGGGGCGTGGATTGAAAT	F	yes	6
P38	P38_NZ_JSVI01000006.1_6	I-B	99,580	101,051	GTTGAACTTTAACATTGGATGTATTTAAAT	F	yes	23
	P38_NZ_JSVI01000107.1_111	I-B	446	1784	GTTGAACTTTAACATTGGATGTATTTAAAT	R	orphan	21
XZL9	XZL9_NZ_ANAP01000038.1_000038	I-B	122	953	GTTTGTATTTTACCTATGAGGAATTGAAAC	R	yes	13
	XZL9_NZ_ANAP01000157.1_000160	I-B	97	1197	GTTTGTATTTTACCTATGAGGAATTGAAAC	F	orphan	17
	XZL9_NZ_ANAP01000220.1_000226	I-B	68	3018	GTTTGTATTTTACCTATGAGGAATTGAAAC	R	orphan	45

## Data Availability

All data generated or analyzed during this study are included in this published article.

## Supplementary Materials

The following supporting information can be downloaded at https://www.mdpi.com/article/10.3390/ijms23063135/s1.
