# Supplementary figures and images for: A Comparative Analysis of Weizmannia coagulans Genomes Unravels the Genetic Potential for Biotechnological Applications
Source: Int J Mol Sci. 2022 Mar 15;23(6):3135. doi: 10.3390/ijms23063135 (PMC8954581; doi:10.3390/ijms23063135)

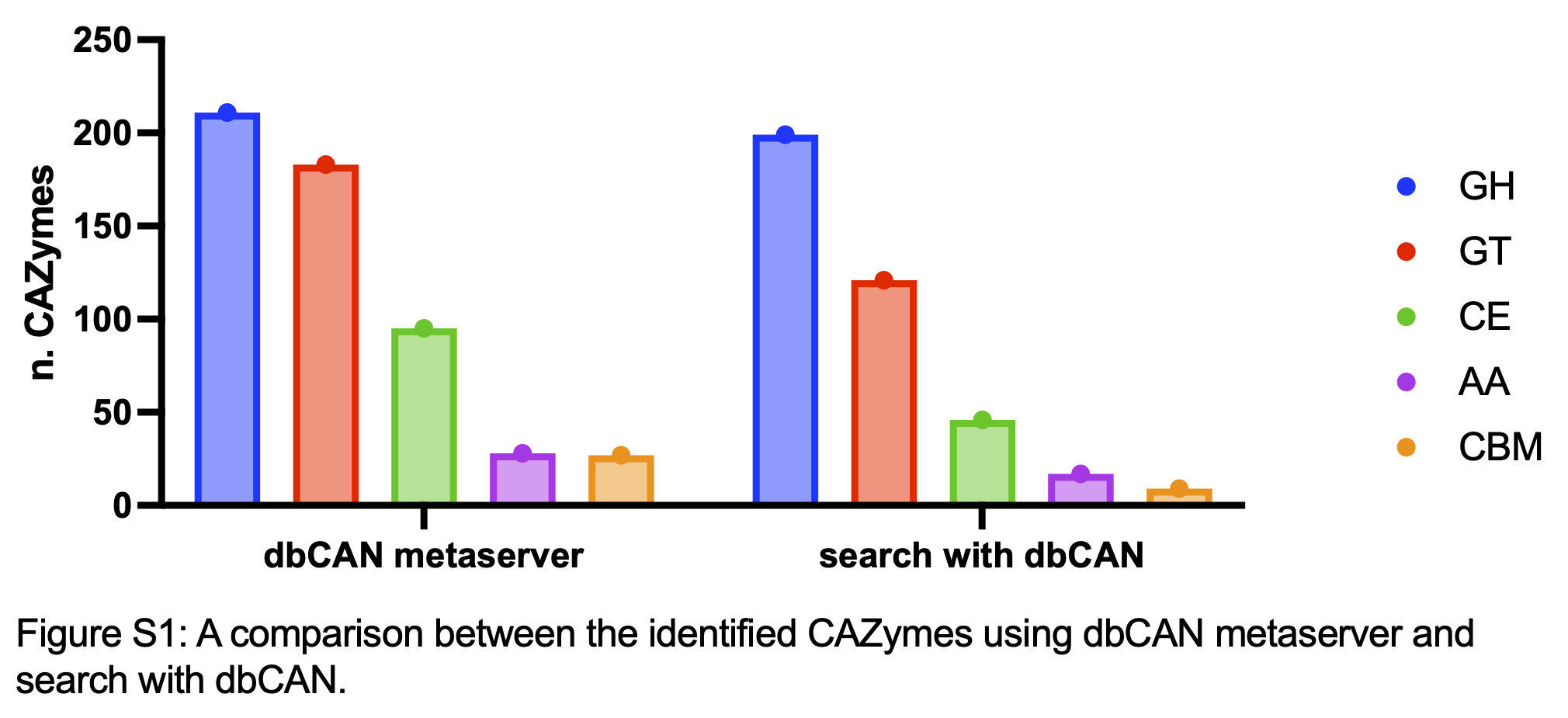

Supplement: Supplementary file 1 [file ijms-23-03135-s001.zip › Figure S1.tiff]
